# Early Parenting Interactions and First-Time Mothers’ Postnatal Depression and Parental Competence

**DOI:** 10.3390/ejihpe14040063

**Published:** 2024-04-06

**Authors:** Rachel W. E. Leong, Davinder Gill, Joanna Barlas, Patrick K. F. Lin

**Affiliations:** School of Social and Health Sciences, James Cook University, 149 Sims Drive, Singapore 387380, Singapore; weienrachel.leong@my.jcu.edu.au (R.W.E.L.); davinder.gill@jcu.edu.au (D.G.); patrick.lin@jcu.edu.au (P.K.F.L.)

**Keywords:** schema therapy, postnatal depression, parental competence, first-time mothers, early interactions

## Abstract

*Objectives*: Schema Therapy, an approach that integrates cognitive-behavioural and attachment principles, helps us understand the impact of early interactions with caregivers on adult mental health. These early interactions can be assessed through Schema Therapy-informed tools; however, these tools have yet to be used with a postnatal population, which represents a period of vulnerability for new mothers. Therefore, the present study aimed to evaluate the impact of positive and negative early parenting interactions on a first-time mother’s mental health and her sense of competence during the postnatal period, using recently revised and newly developed Schema Therapy-informed tools. ***Design***: This is a cross-sectional study. *Method*: First-time mothers (N = 220) participated in an online survey within 12 months post-birth. Participants completed the Positive Parenting Schema Inventory (PPSI), Young Parenting Inventory—Revised (YPI-R2), Edinburgh Postnatal Depression Scale (EPDS), and Parenting Sense of Competence (PSOC) scale. The data were analysed using hierarchical multiple regression and mediational analysis. *Results*: Negative early interactions with mothers and fathers led to greater postnatal depressive symptomology, while positive early interactions with mothers led to fewer postnatal depressive symptoms. Mediation analyses revealed that postnatal depressive symptoms mediated early parenting interactions and participants’ sense of parenting competence as a new mother. *Conclusions*: The protective effects of positive early interactions with caregivers can help first-time mothers’ postnatal emotional adjustment and their sense of competence through diminished postnatal depressive symptoms. However, the enduring effects of negative early interactions with caregivers can contribute to a first-time mother’s risk of developing postnatal depression and negatively affect her sense of parental competence.

## 1. Introduction

Schema Therapy provides us with a framework to both understand and treat the impact of adverse childhood experiences and negative parenting interactions on adult mental health [1,2]. It posits that emotional difficulties in adulthood take root in unmet core emotional needs in childhood through the development of early maladaptive schemas (EMS) [3,4]. EMS arise from an interaction between the child’s temperament and early parenting interactions, which are negative in nature and thus fail to meet the child’s core emotional needs [5]. An EMS is made up of thoughts, feelings, and bodily sensations and becomes a template or pattern through which an individual makes sense of themselves, others, and the world. For example, the EMS of defectiveness is the sense of feeling and believing oneself to be defective, inferior, or unlovable, and typically involves hypersensitivity to criticism or rejection by others. Individuals develop ways of responding to, or coping with, these EMS to either avoid or alleviate the distress that arises when the EMS is triggered. Schema Therapy, through a combination of cognitive, behavioural, experiential, and relational techniques, then seeks to identify and modify these EMS and coping responses to cultivate more adaptive ways of thinking, feeling, and relating to oneself and others [4].

The link between EMS and mental health in adulthood is well established. In addition to numerous individual studies, recent systematic reviews and meta-analyses provide strong evidence for the link between EMS and mental health disorders in adolescence and adulthood [1,6,7,8,9]. For example, all 18 EMS were related to depression in a recent meta-analysis, with schemas associated with feeling flawed, defective, unlovable, and socially excluded being the most strongly associated with depression [6].

The tool used to measure EMS, Young’s Schema Questionnaire-Short Form 3 (YSQ-S3), is one of the most widely used tools from schema theory, translated across multiple languages and with validation studies conducted across several populations [10,11,12,13]. In contrast, tools to measure other relevant schema theory constructs, such as early parenting interactions, are typically less well used and validated [14]. In 2018, the Young Parenting Inventory (YPI) was revised by Louis and colleagues to form the YPI-R2, which was shown to have strong psychometric properties in cross-cultural samples [15]. Additionally, Louis et al. [16] concurrently developed the Positive Parenting Schema Inventory (PPSI), with similarly strong psychometric properties in cross-cultural samples. This shift from a purely psychopathological focus on mental health to considering adaptive functioning is reflective of recent and broader shifts towards positive psychology and wellbeing [17]. The YPI-R2 and PPSI measure negative (‘exasperating’) and positive (‘nurturing’) day-to-day interactions with early caregivers, respectively, which are orthogonal constructs [16]. The quality of these interactions, i.e., whether they exasperate or nurture the child’s core emotional needs, sets the stage for developing psychological health into adolescence and adulthood. An individual who has experienced more exasperating interactions and fewer nurturing interactions in childhood is at greater risk of developing mental health problems in adulthood [18]. The risk increases when stress is present because, although coping responses may have effectively mitigated distress associated with underlying EMS, until that point, they become less effective in stressful situations [4].

First-time motherhood is known to be stressful and, as such, constitutes a vulnerable period for women’s mental health [19,20,21]. It is a challenging transition for women as they navigate issues including postpartum bodily changes, marital difficulties such as negotiating caretaking tasks, and baby care challenges such as understanding the rhythms of the baby’s sleep and the impact on their own sleep [19]. It is thus not uncommon for low mood and postnatal depressive symptoms to arise out of the postpartum period for first-time mothers [21,22,23]. Decades of research reveal risk factors for postnatal depression that span from sociodemographic predictors such as financial hardship and young maternal age to psychosocial predictors such as a history of abuse, as well as limited partner and social support [24,25,26,27,28,29].

Beyond the risk factors that may surround the mother’s experience during her adult years, her experience during her formative years similarly plays a pivotal role in the development of postpartum mood difficulties [30,31]. Commonly examined predictors of postnatal depression include adverse childhood experiences (ACEs) such as childhood abuse [25,30,32], as well as insecure avoidant and anxious attachment styles [31,33]. However, these predictors do not adopt frameworks informed by therapeutic modalities in psychological therapy and are thus, arguably, less clinically useful. The theory underlying Schema Therapy, and particularly the revised YPI-R2 and newly developed PPSI, provides a clinically useful framework to investigate the impact of both positive and negative day-to-day parenting interactions during formative years on first-time mothers’ mental health in the postnatal period.

In addition to the risk of postnatal depression, other aspects of psychological wellbeing, such as a mother’s sense of competence, are likely to be challenged in the postnatal period. Parental competence is used interchangeably with parental self-efficacy in the literature [34,35]. As such, the parental competence discussed in this paper will likewise be grounded in Bandura’s self-efficacy theory [36]. New mothers who feel competent are more likely to utilise effective parenting practices, whereas those who feel less competent may struggle with their parenting, in turn affecting the parent-child relationship, the child’s development, and the mother’s mental health [37]. Furthermore, the choice of parenting practices is known to be, in part, influenced by how an individual was parented via intergenerational transmission of parenting [38]. A first-time mother would draw upon this knowledge, and depending on whether they experienced nurturing or exasperating caregiving in their formative years, this could influence their mood and sense of parental competence differently. Thus, the influence of early parenting interactions can be understood to impact parenting directly and indirectly via mood and feelings of competence.

Several studies have attempted to explore the triadic interrelationships between early parenting interactions, postnatal depression, and parental sense of competence. Adult insecure attachment styles [33] and a history of childhood maltreatment [34] predicted lower parental self-efficacy via the mediating role of maternal depression. These findings suggest negative early childhood experiences play a role in the development of a lower parental sense of competence, particularly through the mediating role of postnatal depression.

## 2. The Present Study

The literature to date implicates the impact of early childhood experiences, using broad constructs that do not sufficiently encapsulate day-to-day interactions with specific caregivers (i.e., mother or father) and do not provide clinically useful frameworks for supporting new mothers who experienced negative interactions with their own caregivers during childhood. Schema theory provides a way of understanding the link between these early interactions and depression in adulthood but has not typically been applied to post-natal depression. The post-natal period is a stressful period for new mothers, and during times of stress, the impact of the negative early interactions is likely to be more pronounced. This is because previous ways of coping may no longer be sufficient to manage the distress associated with the EMS arising out of negative early interactions and because new mothers are likely to be drawing upon the ways in which they were parented, which may leave them feeling low and incompetent. Furthermore, there is a limited focus on the protective role that can be played by nurturing day-to-day interactions with caregivers and how that could be leveraged by postnatal support services. The YPI-R2 and the PPSI provide an opportunity to investigate the impact of both positive and negative day-to-day early parenting interactions on new mothers’ mental health and sense of competence within the framework of schema theory.

Thus, the present study will test the following hypotheses: Firstly, extending on previous studies, it is predicted that negative early interactions will be related to greater postnatal depressive symptomology in first-time mothers, and positive early interactions will be related to lower postnatal depressive symptomology in first-time mothers. Secondly, it is predicted that negative early interactions will be related to a reduced sense of parental competence via greater postnatal depressive symptoms in first-time mothers. In contrast, it is predicted that positive early interactions will be related to an increased sense of parental competence via lower postnatal depressive symptoms in first-time mothers.

Understanding the links between early nurturing parenting interactions, mental health, and a sense of competence will add to our theoretical understanding of the protective role of positive early parenting through a wellbeing lens. This could then provide avenues for reducing the risk or mitigating the impact of postnatal, and potentially intergenerational, mental health problems.

## 3. Methods

### 3.1. Participants

The sample consisted of 220 first-time mothers, aged 18 to 44 years (*M* = 32.04, *SD* = 3.60), with infants between 0 and 12 months (*M* = 5.46, *SD* = 3.22) living in Singapore. All participants completed a series of measures and completed separate ratings for father-related and mother-related early interaction scales (*n* = 212, 218). The predominant nationality of participants was Singaporean, and the predominant ethnic group was Chinese. In terms of socioeconomic information, most participants were employed and had at least a university degree or postgraduate qualification. Lastly, participants’ household income was also observed to be evenly distributed across the income groups. See Supplemental Table S1 for more information.

### 3.2. Design and Procedure

The study employed a cross-sectional research design and data collection through an online anonymous survey administered via Qualtrics^TM^. Participants were recruited online from the community via Facebook interest groups for mothers, postnatal/antenatal support groups, as well as through word of mouth, and they were offered an opportunity to enter a lucky draw upon completion of the survey. The inclusion criteria to participate in this study were: (i) being 18 years of age or older; (ii) primiparous/adoptive mothers with infants under 12 months; (iii) living in Singapore; and (iv) being able to read and write in English. An exclusion criterion was applied to first-time mothers with existing experience with parenting (e.g., with stepchildren). Participants had to consent to the study and complete the whole survey to be included in the final sample. Ethical approval for the study was granted by the Human Research Ethics Committee of James Cook University (HREC Approval Number: H8272).

### 3.3. Measures

Data collected included sociodemographic factors relating to the mother (e.g., mother’s education and employment), the infant (e.g., age), as well as medical history concerning the dead (e.g., birth complications).

The 50-item Positive Parenting Schema Inventory (PPSI) [16] was used to measure recalled positive parenting interactions with participants’ caregivers during childhood. Each item described the participant’s perception of how they were treated (e.g., “Saw me as strong and resilient”). Participants responded to identical items about their father and mother separately, as per the original scale’s instructions and literature showing differences in maternal and paternal parenting styles and practices [39]. Items were scored on a 6-point Likert scale (1 = *completely untrue* to 6 = *describes him/her perfectly*). Positive parenting interaction scores for each parent figure were created by summing all items, with higher scores indicating greater overall nurturing interactions with the respective caregiver. Two scores were derived from the PPSI: positive early interactions with mothers and positive early interactions with fathers. Cronbach’s alpha in our sample was 0.98 for both the mother and father scales.

The 36-item Young Parenting Inventory—Revised (YPI-R2) [15] was used to measure recalled negative parenting interactions with participants’ caregivers during childhood. Each item described the participant’s perception of how they were treated (e.g., “criticised me a lot”). Participants responded to identical items about their father and mother separately. Items were scored on a 6-point Likert scale (1 = *completely untrue* to 6 = *describes him/her perfectly*). Negative parenting interaction scores for each parent figure were created by summing all items, with higher scores indicating greater overall exasperating interactions with the respective caregiver. Two scores were derived from the YPI-R2: negative early interactions with mothers and negative early interactions with fathers. Cronbach’s alpha in our sample was 0.96 and 0.95 for the mother and father scales, respectively. It is noteworthy to point out that the YPI-R2 and PPSI measure orthogonal constructs.

The 10-item Edinburgh Postnatal Depression Scale [40] was used to measure participants’ depressive symptomology during the postpartum period (e.g., “I have been able to laugh and see the funny side of things”). Seven items were reverse-coded. Items were scored on a 4-point Likert scale ranging from 0 (e.g., “*as much as I always could*”) to 3 (e.g., “*not at all*”); however, the labels on the Likert scale varied for each item. Composite scores were created by summing all items, with higher scores indicating greater postnatal depressive symptomology. Cronbach’s alpha in our sample was 0.86.

The 17-item Parenting Sense of Competence scale [41] measured participants’ sense of parental competence during the postpartum period (e.g., “I honestly believe I have all the skills necessary to be a good mother to my child”). Nine items were reverse-coded. All items were scored on a 6-point Likert scale (1 = *strongly disagree* to 6 = *strongly agree*). Composite scores were created by summing all items, with higher scores indicating a greater sense of parental competence. Cronbach’s alpha in our sample was 0.84.

### 3.4. Data Analysis

All statistical analyses were calculated using SPSS 28.0. Data were examined to observe any potential violations of assumptions [42]. One outlier was observed in positive early interactions with mothers; however, as there was no substantive change to results following data analysis with and without the outlier, it was retained to be conservative. Little’s MCAR test was not significant: *X*^2^ (13426, N = 220) = 9970.63, *p* = 1.00, suggesting that data were missing completely at random. Overall, there was less than 5% of missing data, so problems associated with missing data were not considered serious [40]. The mean substitution method was adopted to estimate missing values for data imputation.

Descriptive statistics were computed for sociodemographic variables, and Pearson correlation analysis was run between all demographic variables and dependent variables to identify potential covariates (Table 1). Participants’ age, education level, birth complications, and the baby’s health were identified as potential covariates.

To examine the impact of early parenting interaction on postnatal depressive symptomology, hierarchical multiple regression analyses were conducted. Depressive symptomology was the criterion variable, while negative and positive early interactions with each respective caregiver (mother and father separately) were the predictors. Covariates were entered first, followed by negative early interactions with the respective caregiver, as the relationship between negative parenting interactions and depressive symptomology had been established in existing literature [18,43]. Positive early interactions with the respective caregiver were entered last, as it was a newly explored predictor in the present study. All assumptions about regression were met.

To examine the direct and indirect effects of early parenting interactions on parental competence through postnatal depressive symptomology, mediation PROCESS Model 4 with 5000 bootstrapping samples [44,45,46] was utilised. The respective predictors (i.e., negative or positive early interactions) for each parent figure (mother and father separately) were entered as focal variables into each model, with the identified covariates (i.e., age, education level, birth complications, and the baby’s health) entered to control for their effects. Depressive symptomology was the mediating variable, and parental competence was the dependent variable.

## 4. Results

### 4.1. Impact of Early Interactions with Mothers on Depressive Symptomology

The proportion of variance in depressive symptomology accounted for by covariates and shared variance with early interactions with mothers (both positive and negative) was 16.4% of the overall model *R*^2^, *F* (4, 213) = 10.45, *p* < 0.001. Two covariates negatively predicted depressive symptomology and accounted for unique variance: education level (β = −0.36, *p* < 0.001, 11% of variance) and birth complications (β = −0.13, *p* = 0.049, 2% of variance). The proportion of unique variance in depressive symptomology accounted for by negative early interaction with mothers was 6.4% of the overall model *R*^2^, *F* (1, 212) = 17.61, *p* < 0.001. Thus, negative early interactions with mothers positively predicted depressive symptomology (β = 0.26, *p* < 0.001). Similarly, the proportion of unique variance in depressive symptomology accounted for by positive early interaction with mothers was 1.4% of the overall model *R*^2^, *F* (1, 211) = 3.93, *p* = 0.049. Thus, positive early interactions with mothers negatively predicted depressive symptomology (β = −0.16, *p* = 0.049). Taken together, participants’ education level, birth complications, negative early interactions with mothers, and lack of positive early interactions with mothers all significantly contributed to postnatal depressive symptomology, with 24.2% of the collective variance accounted for in the final model *R*^2^, *F* (6, 211) = 11.25, *p* < 0.001.

### 4.2. Impact of Early Interactions with Fathers on Depressive Symptomology

The proportion of variance in depressive symptomology accounted for by covariates and shared variance with early interactions with fathers (both positive and negative) was 13.8% of the overall model *R*^2^, *F* (4, 207) = 8.32, *p* < 0.001. One covariate negatively predicted depressive symptomology and accounted for unique variance: education level (β = −0.33, *p* < 0.001, 9% of variance). The proportion of unique variance in depressive symptomology accounted for by negative early interaction with fathers was 4.3% of the overall model *R*^2^, *F* (1, 206) = 10.86, *p* = 0.001. Negative early interactions with fathers positively predicted depressive symptomology (β = 0.22, *p* = 0.001). Thus, the hypothesis regarding negative early parenting interactions predicting greater depressive symptomology in first-time mothers was fully supported.

On the contrary, positive early interactions with fathers did not account for any unique variance in depressive symptomology in the overall model *R*^2^, *F* (1, 205) = 0.50, *p* = 0.481. As such, positive early interactions with fathers did not negatively predict depressive symptomology (β = −0.06, *p* = 0.481). Taken together, only education level and negative early interactions with fathers significantly contributed to depressive symptomology, with 18.4% of the collective variance accounted for in the final model *R*^2^, *F* (6, 205) = 7.68, *p* < 0.001. Therefore, the hypothesis regarding positive early parenting interactions predicting lower depressive symptomology was partially supported, in that only positive early interactions with mothers predicted lower depressive symptomology in first-time mothers.

### 4.3. Early Interactions with Mothers and the Mediating Role of Depressive Symptomology

All mediation analyses used PROCESS Model 4 and included age, education level, birth complications, and the baby’s health as covariates. Negative early interactions with mothers (IV) predicted depressive symptomology (MV), *B* = 0.04, *t* = 4.20, *p* < 0.001, but it did not predict parental competence (DV), *B* = −0.03, *t* = −0.147, *p* = 0.144. Depressive symptomology negatively predicted parental competence, *B* = −1.09, *t* = −7.92, *p* < 0.001. After the mediator was entered, the total effect of negative early interactions with mothers on parental competence was significant, *B* = −0.08, *t* = −3.35, *p* = 0.001. Furthermore, the indirect effect of depressive symptomology (MV) was -.05 and significantly excluded zero in the 95% CIs (from −0.07 to −0.02).

Positive early interactions with mothers (IV) negatively predicted depressive symptoms (MV), *B* = −0.03, *t* = −4.17, *p* < 0.001, but it did not predict parental competence (DV), *B* = −0.003, *t* = −0.22, *p* = 0.830. Depressive symptomology negatively predicted parental competence, *B* = −1.16, *t* = −8.35, *p* < 0.001. After the mediator was entered, the total effect of positive early interactions with mothers on parental competence was not significant, *B* = 0.03, *t* = 1.88, *p* = 0.061. The IE of depressive symptomology (MV) was 0.03 and significantly excluded zero in the 95% CIs (from 0.02 to 0.05).

### 4.4. Early Interactions with Fathers and the Mediating Role of Depressive Symptomology

Negative early interactions with fathers (IV) predicted depressive symptoms (MV), *B* = 0.04, *t* = 3.29, *p* = 0.001; however, it did not predict parental competence (DV), *B* = −0.03, *t* = −1.25, *p* = 2.14. Depressive symptomology negatively predicted parental competence, *B* = −1.08, *t* = −7.66, *p* < 0.001. After the mediator was entered, the total effect of negative early interactions with fathers on parental competence was significant, *B* = −0.07, *t* = −2.68, *p* = 0.008. The IE of depressive symptomology (MV) was -.04 and significantly excluded zero in the 95% CIs (from −0.07 to −0.01). Thus, the hypothesis regarding the mediating effect of depressive symptomology between negative early parenting interactions and reduced parental competence was fully supported (Table 2).

Positive early interactions with fathers (IV) negatively predicted depressive symptoms (MV), *B* = −0.02, *t* = −2.34, *p* = 0.020; however, it did not predict parental competence (DV), *B* = 0.02, *t* = 1.41, *p* = 0.161. Depressive symptomology also negatively predicted parental competence, *B* = −1.08, *t* = −7.82, *p* < 0.001. After the mediator was entered, the total effect of positive early interactions with fathers on parental competence was significant, *B* = 0.04, *t* = 2.38, *p* = 0.18. The IE of depressive symptomology (MV) was 0.02 and significantly excluded zero in the 95% CIs (from 0.001 to 0.04). Therefore, the hypothesis regarding the mediating effect of depressive symptomology between positive early parenting interactions and increased parental competence was fully supported (Table 2).

### 4.5. Secondary Analysis

A follow-up analysis was conducted to investigate the discrepant findings between the non-significant relationship of positive early interactions with fathers and depressive symptomology in the hierarchical regression model and the significant relationship of positive early interactions with fathers and depressive symptoms in the mediation model. A hierarchical regression analysis was conducted with depressive symptomology as the criterion, and the predictors and covariates entered in three stages in the following order: positive early interaction with fathers, negative early interactions with fathers, and covariates. The order of variables was adjusted to observe whether the effects of positive early interaction with fathers on depressive symptomology could be explained in the absence of other variables but later eliminated in the presence of variables within a regression model.

The hierarchical multiple regression revealed that at the first stage, positive early interactions with fathers contributed to the regression model, *F* (1, 210) = 12.34, *p* < 0.001, and accounted for 5.6% of the variation in depressive symptomology, which included shared variance with negative early interactions with fathers and the respective covariates. In addition, positive early interactions with fathers negatively predicted depressive symptomology (β = −0.25, *p* < 0.001). This was consistent with the findings in the mediation analysis in the primary analyses. In the subsequent stages of the regression model, however, positive early interactions with fathers did not contribute to or account for unique variance in the overall regression model and were rendered non-significant in their predictive relationship with depressive symptomology (Table 3). This pattern of findings was consistent with the primary analysis’s hierarchical regression model, whereby positive early interactions with fathers were entered in the last stage. As such, it is likely that a relationship exists between positive early interactions with fathers and depressive symptomology; however, it could be considered a weaker variable relative to other variables explored in the present study and thus rendered non-significant when entered last into regression models.

## 5. Discussion

The aim of this study was to examine the impact of early parenting interactions on first-time mothers’ mental health and sense of competence during the postnatal period. Specifically, it used newly revised and developed measures [15,16] derived from Schema Therapy to investigate the impact of negative and positive early parenting interactions on first-time mothers’ postnatal depressive symptomology, as well as the mediating effect of postnatal depressive symptomology on first-time mothers’ sense of competence.

This study’s findings were broadly consistent with previous studies that established risk factors such as adverse childhood experiences (e.g., childhood abuse) and insecure attachment styles (i.e., anxious or avoidant) to be predictive of postnatal depression [25,30,31,32,33]. Specifically, the present study’s findings revealed that negative early interactions with both parent figures were predictive of postnatal depressive symptomology in first-time mothers. This extends on previous studies by highlighting how exasperating parenting interactions, as measured by Schema Therapy-informed tools, with both parents can have lasting impacts by contributing to the development of postnatal mood difficulties in adulthood. Furthermore, findings revealed that positive parenting interactions with mothers and fathers, albeit weaker for the latter, predicted lower postnatal depressive symptomology in first-time mothers. This suggests that the presence of nurturing interactions with mothers serves as a protective factor by reducing the risk of developing postnatal depressive symptoms in new mothers.

In addition, findings also revealed the indirect mediating role of postnatal depressive symptomology on new mothers’ sense of competence. Firstly, negative early interactions with both parents indirectly contributed to a reduced sense of parenting competence in first-time mothers through greater postnatal depressive symptoms. This indicates that exasperating early interactions with a parent in childhood can have negative implications for a new mother in terms of her emotional wellbeing and her sense of competence as a parent, partly due to increased experiences of postnatal depressive symptoms. These findings were consistent with previous studies that found a mediating effect of maternal depression between a history of childhood maltreatment [33] or anxious and avoidant insecure attachment styles [34] and parental self-efficacy. Secondly, positive early interactions with both parents indirectly contributed to increased parental competence in first-time mothers through reduced levels of postnatal depressive symptoms. When there are positive early interactions with either parent, there is a stronger sense of parental competence in taking on the role of a new mother, at least partly due to fewer depressive symptoms during the postpartum period.

From a theoretical perspective, this study supports the use of the schema model with a postnatal population. It provides evidence of the link between early parenting interactions with both mothers and fathers, as defined by schema theory, and mental health and wellbeing in the postnatal period, which is in line with the schema model. The schema model in its entirety would also include temperament, EMS, and coping responses, so this study provides an essential first step for testing the whole model in future research. Understanding the link between both positive and negative day-to-day interactions with early caregivers builds on existing parenting research that Internationalizing parenting using higher-order constructs such as warmth and control [47]. Focusing on day-to-day interactions allows for a more nuanced understanding of parenting interactions that are associated with postnatal depression and, when used clinically, provides a clearer and more precise focus when intervening with mothers who are presenting with, or at risk of developing, postnatal depression.

While this study did not extend to looking at how parenting interactions influenced new mothers’ own parenting styles and the parent-child relationship in the postnatal period directly, previous literature indicates that mothers who feel less competent may struggle to use more effective parenting strategies and, in turn, support their child’s development [37]. Hence, it is proposed that early parenting interactions, as defined in this study, are likely to have an impact on child development across generations, which would also be in line with what is known about intergenerational transmission of parenting [38]. According to schema theory, understanding the specific negative parenting interactions a new mother experienced, as well as the positive interactions she lacked exposure to, helps in identifying her unmet core emotional needs. This could then offer insight into the potential struggles she may face in meeting the core emotional needs of her own child. Understanding which of the parenting interactions used in this study are most susceptible to intergenerational transmission needs to be explored in future research.

### 5.1. Strengths

This study offers several novel contributions. Firstly, most literature conventionally explores early childhood experiences by utilising constructs that are broadly measured (e.g., type of abuse) or through understanding patterns of current close relationships (e.g., adult attachment styles) [25,30,31,32,33]. In the present study, we used two distinct Schema Therapy-related constructs, as measured by the YPI-R2 and PPSI, to measure negative (‘exasperating’) and positive (‘nurturing’) day-to-day interactions with early caregivers. The application of a therapeutic modality framework to understand postnatal psychopathology, in addition to utilising measures derived from it, helps to better inform the formulation and treatment of that psychopathology. In a hypothetical example, a new mother who recalls her own mother as controlling during childhood may remember not being allowed to make mistakes or feeling pressured to meet her assigned responsibilities. These repeated ‘exasperating’ interactions may lead her to believe she is a failure who cannot succeed at things she does (in schema theory, this is an impairment in the domain of autonomy and performance, and the development of a failure schema). Thus, she may be more prone to distressing feelings in situations that leave her feeling like a failure. As she transitions into being a new mother, she will be faced with novel and potentially challenging situations, which may trigger the belief that she has failed at being a mother for not fulfilling her responsibilities to her child. This can lead to increased depressive symptoms and may negatively affect her sense of competence during this transition. An understanding of how controlling interactions with parents increase the risk of postpartum depression could inform a clinician to target the unmet need for autonomy and healthy competence when intervening with this client.

Secondly, postnatal literature also conventionally skews towards examining early experiences with negative constructs. In contrast, the current findings offer a novel viewpoint: nurturing, warm, and caring early interactions with parent figures hold enduring and protective effects on first-time mothers’ postnatal mood adjustment and sense of parental competence. In a similar hypothetical example, a new mother who recalls her own mother as supportive may report that her mother treated her as capable and trusted her ability to make decisions and exercise good judgment. These repeated ‘nurturing’ interactions will likely mean that she develops realistic standards and expectations for herself (in schema theory, this would be the realistic expectations adaptive schema). As she transitions into being a new mother, her realistic expectations of herself enable her to navigate the challenges of motherhood as learning opportunities, as well as self-validate her efforts as “good enough”. As such, she experiences reduced depressive symptomology and may report feeling an increased sense of competence during this transition.

Lastly, the study’s findings also collectively indicate consistent features of intergenerational patterns of parenting. In this sense, the perceptions of historical interactions exchanged between parent and child can have lasting impacts on current parenting behaviour [48]. Moreover, it demonstrates how this further impacts the new mother’s sense of competence through the presence of postnatal depressive symptoms, which may have inadvertent implications for her parenting attitudes and behaviour towards her infant as well.

### 5.2. Clinical Implications

The findings from the present study offer several meaningful clinical implications. In line with existing recommendations, our findings advocate the need for better supporting the mental health of new mothers and offering early interventions to mothers who are struggling with parenting. However, our findings can also offer a more nuanced and specific perspective, given the observed negative impact of negative early parenting interactions in adulthood. Understanding the specific parenting interactions a new mother experienced (or did not experience) provides a guiding framework for Schema Therapy that can be used to address her unmet core emotional needs and the negative impact of EMS on her and her child.

The provisions of post-birth services in Singapore’s public healthcare systems tend to lean towards the baby (e.g., health check-ups), and mothers who are struggling with their mental health would need to seek psychological support through separate adult mental health services. Our findings suggest that Internationalizing a model that concurrently increases a mother’s awareness of positive and negative interactions with her own parents during childhood and helps her to intentionally establish nurturing interactions and reduce exasperating interactions with her own children would benefit the mental health of both mother and child. For example, the Schema Therapy-based early intervention programme Good Enough Parenting [49] and the attachment-based programme The Circle of Security^®^ Parenting^™^ [50,51] aim to prevent the development of psychopathology and improve child attachment outcomes by improving caregiver relational capacities to help parents better understand their own core emotional needs and meet their child’s core emotional needs. While such interventions may initially be seen as costly in terms of the finances and resources required to set them up, evidence shows that early parenting interventions generally can reduce future healthcare and criminal justice system costs via the prevention of child Internationalizing and Internationalizing behaviours [52,53]. Finally, although this study was with postnatal mothers only, the findings highlighted the separate role fathers play in both positive and negative early parenting interactions, thus suggesting that fathers may also need to understand and address their own unmet core emotional needs as they transition to fatherhood; a similar study with postnatal fathers could confirm this.

### 5.3. Limitations

Notwithstanding the abovementioned strengths and clinical implications, there are also limitations to this study that should be considered. Firstly, as positive and negative early interaction scales for parent figures were presented consecutively in a sequential manner, participants may implicitly compare the quality of interactions between mothers and fathers when recalling their past experiences. It was not possible within the design of the present study to administer the parent scales at different time points, as suggested by Tuladhar et al. [48]. However, future studies can consider doing this. In addition, although the present study examined two separate parenting interaction constructs using Schema Therapy-related measures, constructs of Schema Therapy such as early maladaptive schemas were not explored. This was due to the decision to not unduly burden participants with additional survey items since this study was primarily concerned with positive and negative early parenting interactions as measured by the recently revised and developed tools. Given the strong theoretical links between early parenting interactions and the development of early maladaptive schemas on adult psychopathology and parental competence [18,35,43] and the links between exasperating and nurturing parenting interactions now shown in this study, subsequent studies should consider including both adaptive and maladaptive schemas and investigating the relationships between multiple constructs using more complex serial mediation models in PROCESS, or structural equation modelling. Finally, this was a cross-sectional study, and while it is acknowledged that the transition to motherhood is not static, exploring this ongoing transition was beyond the scope of the current study. A longitudinal study would better tap into the impact of early parenting interactions over time as new mothers begin and settle into the journey of motherhood.

### 5.4. Conclusions

Both positive and negative early parenting interactions can impact a new mother’s mental health in the postnatal period. Positive early interactions with her own mother can protect a new mother from depressive symptoms and increase her sense of competence. Conversely, negative interactions with either caregiver can increase the risk of postnatal depression and leave a new mother feeling less competent. Recommendations are made to integrate adult mental health and parenting services to improve healthier intergenerational patterns of parenting.

## Figures and Tables

**Table 1:** Pearson correlations of demographic variables on dependent variables of interest.

	N	Depression	Competence
Age	220	−0.14 *	0.03
Education Level	220	−0.37 **	0.18 **
Birth Complications	220	−0.17 *	0.05
Baby’s Health	220	0.10	−0.20 **

* *p* < 0.05, ** *p* < 0.01.

**Table 2:** Results of mediational analyses on competence via depressive symptomology.

Independent Variable (I.V.)	Bootstrapped Indirect Effects via Depressive Symptoms	95% Confidence Intervals (C.I.)		Total Effects of IV on Competence	Direct Effects of IV on Competence
		Lower Bound (L.B.)	Upper Bound (U.B.)		
Negative early interactions (mothers)	−0.05 (0.01) *	−0.07	−0.02	−0.08 (0.02) *	−0.03 (0.02)
Positive early interactions (mothers)	0.03 (0.01) *	0.02	0.05	0.03 (0.02)	−0.003 (0.01)
Negative early interactions (fathers)	−0.04 (0.02) *	−0.07	−0.01	−0.07 (0.03) *	−0.03 (0.02)
Positive early interactions (fathers)	0.02 (0.01) *	0.001	0.04	0.04 (0.02) *	0.02 (0.01)

Note: Values in brackets are the standard error. * *p* < 0.05.

**Table 3:** Summary of hierarchical regression analysis on depressive symptomology.

Variable	β	t	sr^2^	*R*	*R* ^2^	∆*R*^2^
Step 1				0.24	0.06	0.06
Positive early interactions (fathers)	−0.24 ***	−3.51	0.06			
Step 2				0.30	0.09	0.04
Positive early interactions (fathers)	−0.11	−1.32	<0.01			
Negative early interactions (fathers)	0.23 **	2.90	0.03			
Step 3				0.43	0.18	0.09
Positive early interactions (fathers)	−0.06	−0.71	<0.01			
Negative early interactions (fathers)	0.19 *	2.39	0.02			
Age	0.01	0.08	<0.01			
Education Level	−0.28 ***	−4.02	0.07			
Birth Complications	−0.07	−1.14	0.01			
Baby’s Health	0.10	1.62	0.01			

Note: N = 212. * *p* < 0.05, ** *p* < 0.01, *** *p* < 0.001.

## Data Availability

The data that support the findings of this study are available from the corresponding authors upon reasonable request.

## Supplementary Materials

The following supporting information can be downloaded at: https://www.mdpi.com/article/10.3390/ejihpe14040063/s1, Supplemental Table S1: Sociodemographic characteristics of participants.
