# Supplementary material for: Early Parenting Interactions and First-Time Mothers’ Postnatal Depression and Parental Competence
Source: Eur J Investig Health Psychol Educ. 2024 Apr 6;14(4):963–75. doi: 10.3390/ejihpe14040063 (PMC11049342; doi:10.3390/ejihpe14040063)
Supplement: Supplementary file 1 [file ejihpe-14-00063-s001.zip › ejihpe-2878180-supplementary.pdf]

Supplemental Table S1. Sociodemographic characteristics of participants

	Frequency (N)	Percent (%)
Age		
18 – 24	3	1.4
25 – 29	41	18.6
30 – 34	129	58.6
35 – 39	39	17.7
40 – 44	8	3.6
Ethnicity/Dialect Group		
Hokkien	69	31.2
Teochew	35	15,8
Cantonese	34	15.4
Hakka (Khek)	16	7.2
Hainanese	8	3.6
Malay	27	12.2
Javanese	4	1.8
Tamil	3	1.4
White	5	2.3
Eurasian	3	1.4
Others	16	7.3
Nationality		
Singaporean	182	82.4
Malaysian	19	8.6
Indian	3	1.4
Chinese	3	1.4
Others	13	5.9
Highest Education Level		
Post-secondary of Lower	12	5.5
Polytechnic Diploma	17	7.7
Professional Qualification/Diploma	15	6.8
University (First Degree)	135	61.1
University Postgraduate	41	18.6
Employment Status		
Stay-at-home Mother	32	14.5
Employed	187	84.5
Monthly Household Income		
< S$1000	5	2.3
S$1000 – S$4999	51	23.1
S$5000 – S$8999	60	27.1
S$9000 – S$12999	47	21.3
S$13000 – S$19999	34	15.4
> S$20000	23	10.4
Primary Caregiver (during childhood)		
Mostly mother	120	54.5
Mostly father	5	2.3
Shared equally by mother & father	43	19.5
Foreign domestic worker	15	6.8
Others (e.g., relatives)	37	16.8
Birth Complications (own infant)		
Yes	53	24.0
No	166	75.5
Baby's Health (own infant)		
Excellent	109	49.5
Very Good	82	37.2
Good	23	10.5
Average	6	2.7
